# Antigen Targets for the Development of Immunotherapies in Leukemia

**DOI:** 10.3390/ijms20061397

**Published:** 2019-03-20

**Authors:** Jens Bauer, Annika Nelde, Tatjana Bilich, Juliane S. Walz

**Affiliations:** 1 Department of Hematology and Oncology, University Hospital Tübingen, 72076 Tübingen, Germany; j.bauer@uni-tuebingen.de (J.B.); annika.nelde@uni-tuebingen.de (A.N.); tatjana.bilich@uni-tuebingen.de (T.B.); 2 Institute for Cell Biology, Department of Immunology, University of Tübingen, 72076 Tübingen, Germany

**Keywords:** leukemia, immunotherapy, antigen, target, HLA, epitope, peptide, vaccination, T cell

## Abstract

Immunotherapeutic approaches, including allogeneic stem cell transplantation and donor lymphocyte infusion, have significantly improved the prognosis of leukemia patients. Further efforts are now focusing on the development of immunotherapies that are able to target leukemic cells more specifically, comprising monoclonal antibodies, chimeric antigen receptor (CAR) T cells, and dendritic cell- or peptide-based vaccination strategies. One main prerequisite for such antigen-specific approaches is the selection of suitable target structures on leukemic cells. In general, the targets for anti-cancer immunotherapies can be divided into two groups: (1) T-cell epitopes relying on the presentation of peptides via human leukocyte antigen (HLA) molecules and (2) surface structures, which are HLA-independently expressed on cancer cells. This review discusses the most promising tumor antigens as well as the underlying discovery and selection strategies for the development of anti-leukemia immunotherapies.

## 1. Introduction

Despite promising advances in the understanding of molecular principles and in the treatment of leukemias, these neoplasms still present a substantial health problem in children and adults. Based on data collected in the United States of America from 2011 to 2015, the leukemia incidence rate is indicated with 13.8 new cases and 6.7 leukemia-associated deaths per 100,000 men and women per year (seer.cancer.gov, accessed January 24, 2019). Leukemias are, in general, divisible into a chronic or acute disease type and based on the origin of the malignant cell population in myeloid or lymphoid lineage leading to the classification into four subtypes—chronic lymphocytic leukemia (CLL), acute lymphoblastic leukemia (ALL), chronic myeloid leukemia (CML), and acute myeloid leukemia (AML). Beside these four subtypes there are a number of less common leukemia types, which are not covered in this review.

Within the four main subtypes, CML has a highly specific standard of care. The disease is characterized by the translocation t(9;22) that leads to the formation of the BCR-ABL fusion transcript [1,2]. The resulting fusion protein mediates constitutive tyrosine kinase activity and drives the malignant transformation of CML cells [3]. Inhibition of the BCR-ABL fusion protein by tyrosine kinase inhibitors (TKIs) has led to an impressive improvement in the prognosis of CML patients [4,5,6]. This therapy can achieve a deep molecular response in which the discontinuation of the TKI can be considered; however, several patients suffer from a molecular relapse after a permanent stop of the TKI therapy [7]. Therefore, the standard of care for CML patients includes lifelong TKI therapy, often associated with significant side effects and the risk of developing resistance to TKIs [8,9]. In contrast to CML, the standard treatment for the acute leukemias AML and ALL is dominated by different combinations of chemotherapeutic agents as well as hematopoietic stem cell transplantation. Chemotherapies target in an unspecific manner dividing cells and initially achieve high remission rates; however, this is often followed by acquired drug resistances and high relapse rates especially in AML patients [10,11]. As a result, there is a need for more specific post-remission therapies that are able to target residual chemoresistant malignant cells. In the case of CLL, the addition of CD20-targeting monoclonal antibodies (mABs), e.g., rituximab, to the standard chemotherapy with fludarabine and cyclophosphamide improved the progression-free and overall survival of CLL patients significantly [12,13,14]. The combination of chemotherapy and anti-CD20 mABs comprises the current first-line of treatment for CLL patients after several decades of chemotherapy alone being the standard of care. This highlights the potential of targeted immunotherapies for the treatment of hematological malignancies to achieve long-term progression-free survival.

The immune system has the potential to cure cancers, as evident by the occurrence of spontaneous regression of cancers following infections and the graft-versus-leukemia effect after allogeneic stem cell transplantation in hematological malignancies [15,16]. Immunotherapy is based on the (re)activation of the immune system to attack malignant cells. Non-specific immunotherapy approaches, such as allogeneic stem cell transplantation or interferon-α therapy, have shown to provide long-lasting responses in leukemia patients [17,18]. Furthermore, immune checkpoint inhibitors, which have revolutionized the therapy of many solid tumors in recent years [19,20,21,22,23,24], are currently being evaluated for the treatment of leukemias [25,26]. However, checkpoint inhibitors led to an improved clinical outcome only in a subset of patients and cancer entities, and failed to achieve overall survival benefits in a number of studies [27,28,29,30]. The mutational burden of the cancer and, in case of programmed death-1 (PD-1)-directed checkpoint inhibitors, the programmed death-ligand 1 (PD-L1) expression level on tumor cells have been linked to a favorable outcome of patients treated with checkpoint inhibitors [31,32]. Therefore, further studies are necessary to identify relevant biomarkers in order to select patients, which could benefit from checkpoint inhibitor therapy. Furthermore, the evaluation of suitable combinations of checkpoint inhibitors with conventional chemotherapies, as already successfully proven for lung cancer [33], or antigen-specific immunotherapies in a cancer entity-specific manner might be a promising approach to increase the number of patients that benefit from immune checkpoint inhibition.

**Figure 1:**
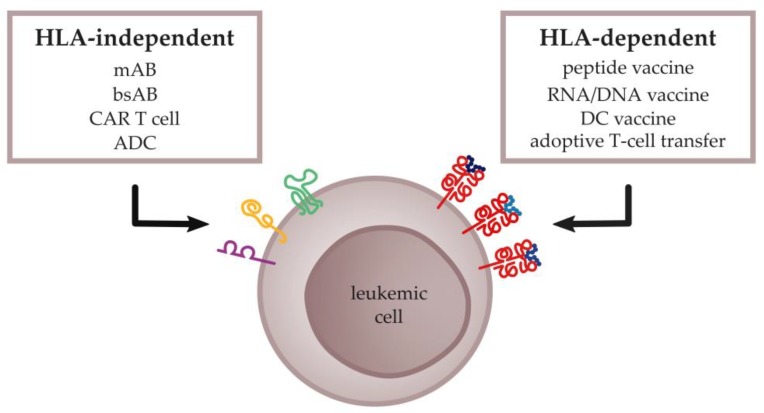
Schematic overview of human leukocyte antigen (HLA)-independent and HLA-dependent antigen-specific cancer immunotherapy approaches. The HLA-independent approach focusses on antibody-based technologies targeting surface structures via e.g., monoclonal antibodies (mABs), bispecific antibodies (bsABs), chimeric antigen receptor (CAR) T cells, or antibody-drug conjugates (ADCs). HLA-dependent immunotherapies induce peptide-specific immune responses mainly by peptide-, RNA/DNA-, or dendritic cell (DC)-based vaccines, and adoptive transfer of activated or T-cell receptor-transduced T cells.

Further advanced immunotherapeutic approaches include agents that induce a more specific immune response against malignant cells, such as vaccines, antibodies, or engineered T cells. The main prerequisite for the development of such antigen-based immunotherapy concepts is the identification of suitable target structures that are specifically expressed on malignant cells. In general, the targets for antigen-specific immunotherapies can be divided into two groups: (1) T-cell epitopes relying on the presentation of peptides via human leukocyte antigen (HLA) molecules and (2) surface structures, which are HLA-independently expressed on cancer cells (Figure 1). This review discusses the most promising tumor antigens for the further development of anti-leukemia immunotherapies.

## 2. HLA-Independent Antigens

Immunotherapeutic approaches targeting HLA-independent antigens are restricted to molecules expressed on the surface of malignant cells. The advantage of these antigens is that they can be represented by any molecule on the cell surface; they are not restricted to peptides bound to an HLA molecule. However, the range of suitable HLA-independent antigens is limited as they have to be exclusively expressed on malignant cells or, if expressed also on benign tissue, at least on partially dispensable tissue to avoid on-target/off-tumor effects. In recent years, different immunotherapy approaches for leukemia, such as mABs, bispecific antibodies (bsABs), antibody-drug conjugates (ADCs), or chimeric antigen receptor (CAR) T cells, have been developed to target cell surface molecules [34,35,36,37,38,39,40]. Such approaches rely on the specific binding to surface antigens. Several B cell-specific surface markers are described, which enable a variety of different immunotherapy approaches to target B-cell malignancies, including CLL and B-ALL. In contrast to these advances, suitable targets, especially for T cell-derived malignancies, are not available, preventing the development of effective targeted therapies so far. The need to identify novel antigens for immunotherapy also holds true for AML and CML patients, because surface molecules expressed on myeloid blasts can also be detected on normal myeloid cells, leaving only a few suitable membrane-expressed antigens that are safe to be targeted by immunotherapy approaches [41].

In the following sections, the most promising antigens for HLA-independent immunotherapy approaches in leukemia are discussed in more detail. In addition to the described targets, there is a variety of other cell surface molecules currently being evaluated in clinical trials and preclinical tests to determine their suitability as targets for immunotherapies, including, for example, BAFF-R, CD23, CD64, CD133, CLL-1, ROR1, and the lambda or kappa light chain of the B-cell receptor (BCR) [42,43,44,45,46,47,48].

### 2.1. CD19

CD19 is a surface molecule commonly expressed on B cells and is thereby a favorable target for B-cell malignancies, such as ALL and CLL. It was first described by Nadler et al. [49], and acts as coreceptor for the B-cell antigen receptor complex. The bsAB blinatumomab and CAR T cells targeting CD19 showed very promising results in ALL and CLL. Both therapies are currently approved for anti-leukemia therapy in patients with ALL [50,51,52]. CD19-directed CAR T cells could achieve complete remissions in up to 90% of patients with relapsed B-ALL [39], whereas durable anti-tumor responses were observed in only 26% of CLL patients infused with CAR T cells targeting CD19 [50]. The most common side effects of CD19-directed immunotherapies are the cytokine-release syndrome, manageable B-cell aplasia, due to the expression of CD19 also on benign B cells, and neurotoxicities in a subset of patients [51,53]. A general problem of immunotherapies targeting a single antigen is epitope loss. In about 10% to 20% of ALL patients, a relapse of CD19-negative leukemic cells was reported after treatment with blinatumomab or CD19-specific CAR T cells [54,55]. The optimal approach to overcome epitope loss is the combination of different antigen targets or therapies. Several combination therapies targeting CD19 together with another specific antigen, such as CD20 or CD22, are currently being evaluated in clinical trials yielding first promising results [40,56].

### 2.2. CD20 and CD22

CD20 and CD22 are both expressed on benign and malignant B cells and are suitable targets for the treatment of B-cell malignancies either as single treatment or as part of a combination therapy. Well-established immunotherapeutic agents in the treatment of CLL targeting CD20 are the humanized murine mABs rituximab and obinutuzumab [12,13,14]. There are several approaches of combination therapies targeting CD19 and CD20 to prevent antigen loss. For example, CAR T cells expressing an anti-CD19/CD20 bispecific receptor, which prevented antigen escape of malignant B cells in lymphoma cell lines [40].

CD22 is a sialoglycoprotein and part of the immunoglobulin superfamily. When CD22 is bound by a mAB it gets rapidly internalized, qualifying it as an excellent target for ADC-based immunotherapy [57]. Inotuzumab ozogamicin, an ADC comprised of a humanized mAB against CD22 conjugated to the cytotoxic agent calicheamicin, led to a higher progression-free and overall survival in B-ALL patients compared to the standard therapy [58]. CD22-targeting CAR T cells induced remissions in B-ALL patients that were either naïve or developed resistance to CD19-specific immunotherapy [59]. Furthermore, the development of anti-CD19/CD22 bispecific CAR T cells showed first promising results in ALL cell line xenograft and patient-derived xenograft experiments [56].

### 2.3. CD33

The CD33 antigen, also named sialic acid-binding Ig-like lectin 3 (Siglec-3), is expressed on the cell surface of myeloid cells and plays a role in mediating cell-cell interactions. This marker is expressed on AML blasts in up to 90% of the cases and on AML progenitor cells, but also on benign myeloid cells [60,61]. This complicates targeting CD33 due to myelosuppression in patients treated with CD33-targeted immunotherapies [38,62]. The ADC gemtuzumab ozogamicin was the first approved targeted compound for CD33. It was granted accelerated approval by the FDA in the year 2000 following a promising phase II study; however, a phase III study revealed considerable liver toxicity and the drug was voluntarily withdrawn after it failed to show a survival benefit compared to chemotherapy [63]. In 2017, the FDA reapproved gemtuzumab ozogamicin for newly diagnosed and relapsed/refractory AML patients due to novel findings in clinical trials which applied different doses and different schedules of the drug [64]. CAR T cells against CD33 displayed significant effector functions in vitro, but reduction of myeloid progenitors in xenograft models and one AML patient suggested that persistence of CD33-specific CAR T cells entails huge hematopoietic toxicity [62,65]. In contrast, AMG 330, a CD3/CD33-targeting bsAB, showed promising preclinical data, suggesting it as a potential future therapy for AML [36].

### 2.4. CD123

Interleukin-3 receptor subunit alpha or CD123 is very frequently expressed on both blast and leukemic progenitor cell populations in AML as well as in B-ALL and other rare leukemia subtypes, but not in T-ALL [66]. Due to the important role of interleukin-3 signaling in hematopoietic development, CD123 is also present on hematopoietic stem/progenitor cells; however, in contrast to CD33, CD123 shows only low to negligible expression on corresponding benign cells [67]. This differential expression pattern predefines CD123 as an attractive target. So far, immunotherapeutic approaches against CD123 revealed tolerable toxicity and promising anti-leukemia effects in preclinical and phase I studies [35,37,68,69]. The combination of CD123- and CD19-directed CAR T cells could demonstrate an improved outcome in xenograft models and holds the potential to overcome CD19 epitope loss in B-ALL [70].

### 2.5. FMS-Like Tyrosine Kinase-3 (FLT3)

The receptor tyrosine kinase FMS-like tyrosine kinase-3 (FLT3) plays an important role in the development of the hematopoietic and the immune system [71]. FLT3 is expressed on the cell surface of AML blasts and approximately one-third of AML patients carry internal tandem duplications (ITDs) in the FLT3 gene, which are associated with a poor prognosis [72]. The FLT3-ITD mutations lead to conformational changes of the receptor and thereby to constitutive autophosphorylation and induction of proliferation [73]. This role in disease development underlines the potential of FLT3 as a suitable target in AML patients. Potent reactivity of CAR T cells targeting FLT3 was demonstrated against AML cell lines and primary AML blasts in mouse models [74]. The mAB FLYSYN targeting mutated and unmutated FLT3 is currently being evaluated in a clinical study [34]. Additionally, a bsAB targeting FLT3 and CD3 was developed on the basis of the FLYSYN mAB and exhibited enhanced cellular cytotoxicity against FLT3-expressing AML cells in comparison to the mAB alone [75]. A combination of TKIs and the FLT3-targeting bsAB also revealed promising results, explained by an increased cell surface localization of FLT3-ITD after TKI treatment in xenograft models with blast cells from FLT3-ITD-positive AML patients [76].

## 3. HLA-Dependent Antigens

The presentation of peptides bound to HLA molecules on the surface of a cell comprises the core of the antigen presentation machinery of the immune system. The entirety of HLA-presented peptides is referred to as the immunopeptidome. HLA class I or class II molecules present peptides to CD8^+^ or CD4^+^ T cells, respectively, and can thereby trigger the eradication of malignant or infected cells. Leukemia-specific HLA epitopes can be exploited for immunotherapy approaches to induce anti-leukemia T-cell responses. One main advantage of the HLA-dependent presentation of antigens is that these antigens can also originate from intracellular proteins and are not restricted to surface proteins. Therefore, the number of potential targets is considerably higher, which facilitates the identification of new antigens. However, the HLA allotype restriction of the peptide targets represents a limitation concerning the development of broadly applicable immunotherapy approaches and calls for the combination of peptides of various HLA restrictions or for patient (group)-individualized approaches. Several strategies such as peptide, DNA/RNA, or dendritic cell (DC) vaccination approaches have been developed to target tumor-specific T-cell epitopes. These studies clearly showed that such vaccination approaches are able to induce T-cell responses and are furthermore well tolerated with only minor side effects [77,78,79]. Moreover, HLA epitopes can also be targeted by specific antibodies or by adoptive T-cell transfer using T cells transduced with a known T-cell receptor (TCR) against a single tumor-associated antigen [80,81].

### 3.1. Identification Methods

**Figure 2:**
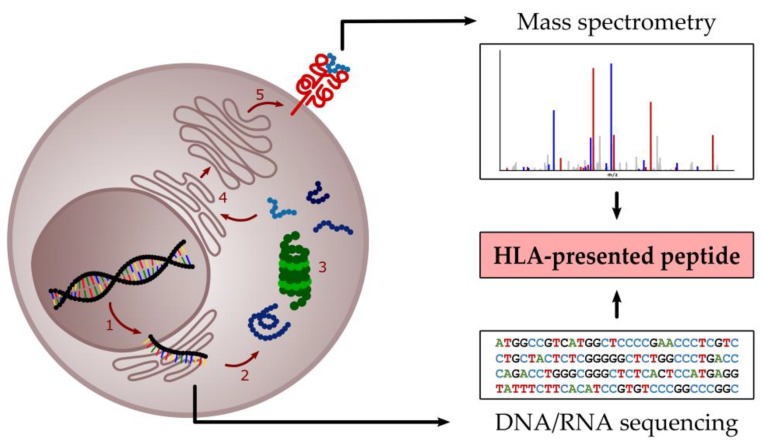
Schematic overview of the immunopeptidome-centric approach and the gene expression-based reverse immunology approach for the identification of HLA-presented peptides as targets for anti-cancer immunotherapy. A simplified depiction of the cellular processes involved in HLA antigen processing is illustrated, including (1) DNA transcription, (2) protein biosynthesis, (3) proteasomal degradation, and (4) peptide loading on HLA molecules via the endoplasmic reticulum and the Golgi apparatus, resulting in (5) the cell surface presentation of the HLA-peptide complex. The direct identification of naturally presented HLA-restricted peptides is based on the isolation of HLA-peptide complexes, followed by peptide purification, and peptide sequence identification by liquid chromatography-coupled tandem mass spectrometry (LC-MS/MS). In contrast, the reverse immunology approach is based on DNA and/or RNA isolation and sequencing, followed by in silico epitope prediction of mutation-derived or overexpressed proteins.

There are basically two different approaches for the identification of suitable HLA-restricted target antigens for immunotherapy—a gene expression-based so-called reverse immunology approach and an immunopeptidome-centric approach as illustrated in Figure 2. The reverse immunology approach focuses on genome/exome and/or RNA sequencing of malignant cells in comparison to a healthy control with the aim of identifying non-synonymous mutations or upregulated proteins, which are specific for the malignant cells. In order to determine potential HLA ligands derived from these mutated or upregulated proteins, an in silico prediction is performed followed by further validation steps and immunogenicity analyses of the predicted epitopes in T-cell assays [82,83,84]. This method has several limitations for the identification of suitable targets for anti-leukemia immunotherapy. First, there is no direct correlation between the genome, the transcriptome, the proteome, and the naturally presented immunopeptidome [85,86,87,88,89], thus making assumptions based solely on gene expression data inconclusive. Nevertheless, the gene expression-based approach plays an important role for the identification of tumor-specific mutated genes that could lead to the presentation of so-called neoantigens, which are known to play a major role in immune responses against cancers, especially in the context of immune checkpoint inhibitors [90,91,92,93,94,95]. However, several studies provide evidence that only a very small fraction of the genomic mutations in a cancer cell is presented as HLA ligand on the cell surface [93,95,96,97]. Exemplarily, a personalized vaccination study for newly diagnosed glioblastoma patients (*n* = 15) identified a total of 643 genomic mutations, but could confirm none of them in the HLA class I and II immunopeptidome of the corresponding patients [98]. Another study searching for neoantigen-derived HLA ligands in melanoma patients, a cancer entity bearing one of the highest mutational burdens [91], detected in five patients with a high number of non-synonymous mutations (>15,000 per tumor sample) only 11 naturally presented neoepitopes [87]. This data suggests a minor role of genome sequencing-based neoantigen predictions for the treatment of leukemias, which are known as low mutational burden malignancies [91].

The immunopeptidome-centric approach focuses on the direct identification of naturally presented HLA-restricted peptides on malignant cells [99]. Therefore, HLA-peptide complexes are isolated from lysed cells by immunoaffinity purification with HLA-specific antibodies and subsequently analyzed by liquid chromatography-coupled tandem mass spectrometry (LC-MS/MS) [86,100,101,102,103,104,105,106]. To identify leukemia-exclusive HLA ligands, the immunopeptidomes of malignant cells and benign samples from healthy donors are comparatively analyzed. Exclusive or strongly upregulated ligands are then further analyzed in T-cell assays to determine their capacity to induce peptide-specific T-cell responses [101,104,107]. Technological advances in recent years enable comprehensive mapping of the immunopeptidome landscape of primary patient material in unprecedented depth, which, in turn, allows for the implementation of novel strategies of antigen identification based solely on HLA ligandome data [87,98,101,103,104,108]. This is, so far, the only unbiased methodology to comprehensively analyze the naturally presented HLA-peptide repertoire and might, therefore, represent a highly effective and indispensable method for the identification of immunologically relevant tumor antigens [109].

### 3.2. HLA-Presented Peptide Targets

In recent years, a considerable number of leukemia-associated antigens (LAAs) have been described and will be discussed in detail in the following subsections. Several of these LAAs showed promising results in preclinical and clinical studies for their use in immunotherapy approaches. An overview of currently ongoing clinical studies based on HLA-presented peptide targets in leukemia patients is set out in Table 1. An important point, which must be considered, concerning the selection of HLA-presented LAAs, is that tumor-exclusivity can either be assessed on the level of HLA ligands or on the level of the entire antigen. Single HLA ligands from one protein can be tumor-exclusive even if other peptides from the same antigen are also presented on benign cells. This fact could be explained by different splicing, protein modifications, or antigen processing in cancer cells, which lead to an altered presentation of the immunopeptidome compared to benign cells [104]. Therefore, the Tübingen approach was developed to identify immunotherapeutic relevant HLA ligands. In a first step, naturally presented HLA-restricted peptides are directly identified from primary tumor cells using the LC-MS/MS technology. Next, identified tumor-associated peptides are selected by differential gene expression analysis, data mining, and most importantly, comparative analysis with the ligandome of benign cells. In a last step, selected candidates are validated by in vitro T-cell assays and, where possible, monitoring in vivo T-cell responses in the context of patient-individualized immunizations [110]. Studies following this approach allow, on the one hand, the development of broadly applicable off-the-shelf immunotherapies targeting non-mutated LAAs, especially for malignancies with low mutational burden including leukemias [86,104,108], and on the other hand, the design of personalized peptide-based immunotherapies based on the patient-individual immunopeptidome analysis of tumor cells [111]. A recently conducted meta-analysis of the HLA peptidome composition in different hematological entities revealed that there is only a small amount of entity-spanning antigens, suggesting that the design of peptide-based immunotherapies for the treatment of hematological malignancies should ideally be realized in an entity-specific manner [112].

The following subsections provide insights into the most promising antigen targets for novel immunotherapy approaches targeting leukemic cells in an HLA-dependent manner.

#### 3.2.1. Wilms’ Tumor Gene (WT1)

Wilms’ tumor gene (WT1) encodes a transcription factor, which was originally identified as mutated in patients with Wilms’ tumor, a nephroblastoma that typically occurs in children. WT1 was described early as widely expressed in AML, ALL, and CML, but not in CLL [113]. Expression of WT1 on normal hematopoietic progenitor cells was reported as at least 10 times less than in AML cells, confirming the overexpression of the WT1 gene in leukemic cells [114]. Quantification of WT1 gene expression can be used to predict the probability of relapse and to detect minimal residual disease in AML patients [115,116]. Interestingly, WT1 was ranked as the top immunotherapy target in cancer by a national cancer institute pilot project for the prioritization of cancer antigens [117]. Numerous clinical studies were carried out implementing peptide vaccination strategies for AML patients in remission after an initial chemotherapy and demonstrated that they are able to induce T-cell responses and are well tolerated with only minor side effects [79,118,119,120]. Furthermore, DC vaccination and adoptive transfer of WT1-specific cytotoxic T cells showed to be auspicious therapeutic options in combination with allogeneic stem cell transplantation in AML patients [121,122].

#### 3.2.2. Telomerase

One of the most important proteins for cancer development and progression, also titled the “universal tumor-associated antigen”, the human telomerase reverse transcriptase (hTERT) is up-regulated or reactivated in approximately 90% of human cancers [123]. In healthy individuals, hTERT maintains telomere length in stem and germ cells but is usually silenced in almost all somatic cells. The first identified immunogenic peptide from hTERT was I540 (ILAKFLHWL) restricted to HLA-A*02 [124]. Since then, several peptides of hTERT have been described as immunogenic and increasing evidence suggests that the protein can serve as a suitable target for widely applicable immunotherapy approaches against cancer. A DC vaccination study targeting hTERT induced in 11 out of 19 AML patients hTERT-specific T-cell responses and 64% of the responders were free of disease recurrence at the time of their last follow-up with a median follow-up of 52 months [125]. In humanized mouse models, adoptive T-cell transfer utilizing T cells transduced with a TCR recognizing an HLA-A*02-restricted hTERT-derived peptide were able to control human B-ALL, CLL, as well as AML progression in vivo, supporting the feasibility of hTERT-directed adoptive immunotherapy [126,127].

#### 3.2.3. Receptor for Hyaluronan-Mediated Motility (RHAMM)

CD168 or receptor for hyaluronan-mediated motility (RHAMM) was described as widely expressed LAA in AML, CML, and CLL [128,129]. A possible role of RHAMM as a biomarker for patient prognosis in acute pediatric leukemias was investigated revealing that patients with a high percentage of RHAMM-positive blast cells at the time of diagnosis had more blasts in minimal residual disease and a poorer prognosis [130]. By the in silico prediction of potential HLA-A*02-presented peptides from RHAMM, the peptide R3 (ILSLELMKL) was first characterized as a highly immunogenic CD8^+^ T-cell epitope in AML patients [83]. A phase I clinical trial assessed the efficacy of a peptide vaccination with RHAMM R3 in patients with AML, myelodysplastic syndrome (MDS), and multiple myeloma and showed in 7 out of 10 patients an increase of RHAMM R3-specific effector T cells with three patients achieving clinical responses [131]. Another study with RHAMM R3 was conducted in six CLL patients and showed similar promising results [132]. Interestingly, it was described that RHAMM is already expressed and presented by monocyte-derived DCs from AML patients in a DC vaccination setting, even without RHAMM mRNA electroporation, and that this is sufficient to activate RHAMM-specific T cells [133]. On the contrary, a study by Schauwaert et al. concluded that RHAMM is a suboptimal target antigen for immunotherapy approaches in AML patients, due to an equal expression level of RHAMM in leukemic stem cells and hematopoietic stem cells from healthy controls indicating that an elimination of leukemic stem cells is unlikely to be achieved by RHAMM-directed immunotherapy [134].

#### 3.2.4. Preferentially Expressed Antigen of Melanoma (PRAME)

The antigen PRAME (preferentially expressed antigen of melanoma) was initially identified in two melanoma cell lines and was described to be overexpressed in a large fraction of different tumors and leukemias. PRAME is a classical cancer-testis antigen and is considered a highly attractive target in leukemias. Multiple HLA-A*02-restricted epitopes of PRAME e.g., VLDGLDVLL, SLYSFPEPEA, ALYVDSLFFL, and SLLQHLIGL have been described and showed spontaneous CD8^+^ T-cell reactivity in ALL, AML, and CML patients [135]. The ALYVDSLFFL/HLA-A*02 complex can also be targeted by the TCR mimic mAB Pr20, which was therapeutically effective in mouse xenograft models of human leukemias [136]. Another study identified PRAME peptide-specific CD8^+^ effector and effector memory T cells in healthy individuals, which suggests PRAME as the preferable LAA for adoptive T-cell therapies focusing on the generation of LAA-specific T cells from healthy allogeneic HLA-matched T-cell donors [137].

#### 3.2.5. PR1

The PR1 epitope is a nonameric peptide (VLQELNVTV) in complex with an HLA-A*02 molecule and is recognized by cytotoxic T lymphocytes when it is presented on the surface of CML or AML cells in patients. It originates from the two serine proteases proteinase 3 (P3) and neutrophil elastase (NE) that are overexpressed and mislocalized in myeloid leukemia blasts [138]. A peptide vaccination study, including 66 patients with CML, MDS, or AML, showed that PR1 induced specific immunity correlated with clinical responses, including molecular remission [139]. Furthermore, the novel developed T-cell receptor-like mAB 8F4 binds with high affinity a combined epitope of the PR1/HLA-A*02 complex and induced complement-dependent cytolysis of AML blasts and Lin^−^CD34^+^CD38^−^ leukemic stem cells, but not of normal leukocytes [140]. The Molldrem group also developed a bsAB binding the PR1/HLA-A*02 complex and CD3 to improve the potency of the 8F4 mAB. This bsAB could activate T cells to lyse HLA-A*02^+^ primary AML blasts and cell lines [141]. Additionally, a TCR-like CAR with specificity for the PR1/HLA-A*02 complex, on the basis of the 8F4 mAB, was constructed, and transduced CAR T cells were capable to kill leukemia cell lines and primary AML blasts in vitro in an HLA-A*02-dependent manner [142].

#### 3.2.6. Survivin

The survivin protein, also known as baculoviral IAP repeat-containing protein 5 (BIRC5), is an inhibitor of apoptosis and promotes cell survival. Survivin gene expression was proven in ALL, AML, and CML blasts, but not in normal bone marrow cells. Additionally, the disease-free survival rates of patients with survivin expression were lower than that of patients without survivin expression [143]. In a proof of concept study, using a xenograft mouse model of primary ALL, the knockdown of survivin by shRNA in combination with chemotherapy resulted in no detectable minimal residual disease, demonstrating that targeting survivin can overcome chemotherapy resistance [144]. In an expression analysis effort of adult B-ALL patients, survivin was the only LAA that was identified to be significantly overexpressed in these patients compared to healthy donor samples, suggesting survivin as an excellent target antigen for immunotherapy in adult B-ALL patients [145].

#### 3.2.7. BCR-ABL

Philadelphia-positive leukemias, including CML and some ALL cases, possess the BCR-ABL fusion protein, which originates from the chromosome translocation t(9;22). This translocation results in novel BCR-ABL fusion regions and peptides originating from these breakpoints can be classified as leukemia-specific neoantigens. Therefore, the BCR-ABL fusion region is an attractive leukemia-specific T-cell target. Furthermore, most patients with a Philadelphia-positive leukemia receive TKI therapy, which can lead to additional mutations in the BCR-ABL fusion protein in order to achieve resistance to the inhibitor, which in turn could lead to new neoantigens naturally presented on the cell surface. By the use of epitope prediction, several immunogenic BCR-ABL fusion region peptides were described, which could induce BCR-ABL-specific CD4^+^ and CD8^+^ T-cell responses [146,147,148]. These T cells were capable of controlling treatment-refractory Philadelphia-positive ALL in vivo after adoptive T-cell therapy [146]. An in vitro study, which computationally predicted new TKI therapy-induced BCR-ABL neoantigens, could demonstrate specific CD8^+^ T cells against a HLA-A*03-restricted peptide in the blood of two CML patients [149]. The BCR-ABL-specific HLA-A*03-restricted peptide KQSSKALQR was reported as identified in one patient by LC-MS/MS [150]. However, a recent study searching for BCR-ABL- and ABL-BCR-derived neoepitopes in 21 CML patients, including eight HLA-A*03-positive patients, could not detect any naturally presented neoepitopes by LC-MS/MS analysis [104]. This indicates that these epitopes are rare and technically challenging to confirm.

#### 3.2.8. NPM1

C-terminal mutation in nucleophosmin (NPM1) defines the most frequent mutation in AML and is associated with a favorable prognosis of patients [151]. This beneficial outcome of NPM1-mutated AMLs is linked to the recognition of the NMP1 mutation by the immune system leading to CD4^+^ and CD8^+^ T-cell responses in patients [152,153]. Furthermore, an association of HLA class I genotypes with the prevalence and outcome of patients with AML and mutated NPM1 was reported, indicating the importance of peptide presentation on certain HLA molecules [154]. In a recent study, researchers isolated CD8^+^ T cells recognizing the LC-MS/MS-validated HLA-A*02-restricted neoepitope CLAVEEVSL [155]. Additionally, they isolated the epitope-specific TCR and demonstrated in a xenograft model the capacity of TCR-transduced T cells to lyse AML cells expressing mutated NPM1. All these findings suggest NPM1 as an excellent target antigen for HLA-dependent immunotherapy approaches in AML patients with NPM1 mutations.

## 4. Conclusions

Antigen-specific immunotherapy is a rapidly growing field providing promising new therapeutic options in virtually all areas of cancer therapy, including leukemias. Checkpoint inhibitors, monoclonal and bispecific ABs, adoptive T-cell transfer, and vaccination approaches hold the potential to improve the prognosis and survival of leukemia patients. To further increase the number of patients that benefit from antigen-specific immunotherapies, the characterization of suitable target antigens, fulfilling the following criteria, is indispensable: (1) Tumor exclusivity, the antigen should be naturally presented and only expressed on malignant cells to minimize on-target/off-tumor effects in patients; (2) immunogenicity, the naturally presented epitope should be recognized by T cells in vivo; (3) broad expression, the optimal target should be highly expressed in a majority of patients or even spanning several cancer entities.

HLA-independent targets exhibit the advantage that they are broadly applicable for many patients due to lineage-specific expression patterns, but the variety of possible antigens that are exclusively expressed on cancer cells is limited. In contrast, there is a large variety of tumor antigens presented by HLA molecules with the limitation of the restriction to a specific HLA allotype. However, a single optimal tumor antigen for all cancer entities might not exist. Targeting several antigens seems beneficial in terms of increasing specificity and minimizing the possibility of antigen loss and therapy resistance of malignant cells. Therefore, a combination of antigens and targeting modalities might be the future for clinically effective cancer immunotherapy. This includes, for example, the combination of antigen-specific therapies with unspecific therapies such as immune checkpoint inhibitors or cytokines for the achievement of synergistic effects from various immunotherapeutic agents [93,156,157]. Moreover, the optimal combination of immunotherapies, especially vaccination-based approaches, with suitable adjuvants, including, for example, Toll-like receptor (TLR) agonists [158,159], has to be further evaluated in order to boost the efficacy of cancer immunotherapies [160]. Over the last decade, the methodology for antigen detection, as well as for effective targeting of tumor antigens, has experienced a quantum leap, providing us all necessary tools required for an effective anti-leukemia immunotherapy. Future studies are now facing the challenging task to select and combine these antigens and immunotherapeutic approaches to design an effective treatment for each individual leukemia patient.

## Figures and Tables

**Table 1:** Current clinical trials focusing on HLA-dependent immunotherapy in leukemia.

Identifier	Treatment	Target	Disease	Phase	Status
NCT03083054	DC vaccination	WT1	AML, MDS	I/II	recruiting
NCT02405338	DC vaccination	WT1, PRAME	AML	I/II	active, not recruiting
NCT02543749	DC vaccination	BCR-ABL, WT1, PR3	CML	I/II	recruiting
NCT01686334	DC vaccination	WT1	AML	II	recruiting
NCT03291444	DC vaccination + CAR T cells	WT1, Eps8	ALL, AML, MDS	I	recruiting
NCT03265717	DNA vaccination	hTERT	CLL	II	recruiting
NCT02802943	peptide vaccination	patient-individualized	CLL	II	recruiting
NCT03559413	peptide vaccination	patient-individualized	ALL	I/II	recruiting
NCT02750995	peptide vaccination + azacitidine	NY-ESO-1, PRAME, MAGE-A3, WT1	AML, MDS	I	recruiting
NCT02494167	adoptive T-cell transfer	WT1, NY-ESO-1, PRAME, Survivin	AML, MDS	I	recruiting
NCT02475707	adoptive T-cell transfer	WT1, PRAME, Survivin	ALL	I	recruiting
NCT00620633	adoptive T-cell transfer	WT1	leukemia, MDS	I	active, not recruiting
NCT03326921	TCR-transduced adoptive T-cell transfer	HA-1	ALL, AML	I	recruiting
NCT02770820	TCR-transduced adoptive T-cell transfer	WT1	AML	I/II	recruiting
NCT02743611	TCR-transduced adoptive T-cell transfer	PRAME	AML, MDS, uveal melanoma	I/II	recruiting

## Abbreviations

ADC	Antibody-drug conjugate
ALL	Acute lymphoblastic leukemia
AML	Acute myeloid leukemia
BCR	B-cell receptor
bsAB	Bispecific antibody
CAR	Chimeric antigen receptor
CLL	Chronic lymphocytic leukemia
CML	Chronic myeloid leukemia
DC	Dendritic cell
FLT3	FMS-like tyrosine kinase-3
HLA	Human leukocyte antigen
hTERT	Human telomerase reverse transcriptase
ITD	Internal tandem duplication
LAA	Leukemia-associated antigen
LC-MS/MS	Liquid chromatography-coupled tandem mass spectrometry
mAB	Monoclonal antibody
MDS	Myelodysplastic syndrome
NPM1	Nucleophosmin
PRAME	Preferentially expressed antigen of melanoma
PD-1	Programmed death-1
PD-L1	Programmed death-ligand 1
RHAMM	Receptor for hyaluronan-mediated motility
TCR	T-cell receptor
TLR	Toll-like receptor
TKI	Tyrosine kinase inhibitor
WT1	Wilms’ tumor gene

## References

[1] Nowell P.C., Hungerford D.A. (1960). Chromosome studies on normal and leukemic human leukocytes. J. Natl. Cancer Inst..

[2] Brehme M., Hantschel O., Colinge J., Kaupe I., Planyavsky M., Köcher T., Mechtler K., Bennett K.L., Superti-Furga G. (2009). Charting the molecular network of the drug target Bcr-Abl. Proc. Natl. Acad. Sci. USA.

[3] Daley G., van Etten R., Baltimore D. (1990). Induction of chronic myelogenous leukemia in mice by the P210bcr/abl gene of the Philadelphia chromosome. Science.

[4] O’Brien S.G., Guilhot F., Larson R.A., Gathmann I., Baccarani M., Cervantes F., Cornelissen J.J., Fischer T., Hochhaus A., Hughes T. (2003). Imatinib compared with interferon and low-dose cytarabine for newly diagnosed chronic-phase chronic myeloid leukemia. N. Engl. J. Med..

[5] Cortes J.E., Kim D.-W., Kantarjian H.M., Brümmendorf T.H., Dyagil I., Griskevicius L., Malhotra H., Powell C., Gogat K., Countouriotis A.M. (2012). Bosutinib versus imatinib in newly diagnosed chronic-phase chronic myeloid leukemia: Results from the BELA trial. J. Clin. Oncol..

[6] Kantarjian H.M., Kim D.-W., Pinilla-Ibarz J., Le Coutre P.D., Paquette R., Chuah C., Nicolini F.E., Apperley J., Khoury H.J., Talpaz M. (2014). Ponatinib (PON) in patients (pts) with Philadelphia chromosome-positive (Ph+) leukemias resistant or intolerant to dasatinib or nilotinib, or with the T315I mutation: Longer-term follow up of the PACE trial. J. Clin. Oncol..

[7] Saussele S., Richter J., Guilhot J., Gruber F.X., Hjorth-Hansen H., Almeida A., Janssen J.J.W.M., Mayer J., Koskenvesa P., Panayiotidis P. (2018). Discontinuation of tyrosine kinase inhibitor therapy in chronic myeloid leukaemia (EURO-SKI): A prespecified interim analysis of a prospective, multicentre, non-randomised, trial. Lancet Oncol..

[8] Schmidt M., Rinke J., Schäfer V., Schnittger S., Kohlmann A., Obstfelder E., Kunert C., Ziermann J., Winkelmann N., Eigendorff E. (2014). Molecular-defined clonal evolution in patients with chronic myeloid leukemia independent of the BCR-ABL status. Leukemia.

[9] Machova Polakova K., Kulvait V., Benesova A., Linhartova J., Klamova H., Jaruskova M., de Benedittis C., Haferlach T., Baccarani M., Martinelli G. (2015). Next-generation deep sequencing improves detection of BCR-ABL1 kinase domain mutations emerging under tyrosine kinase inhibitor treatment of chronic myeloid leukemia patients in chronic phase. J. Cancer Res. Clin. Oncol..

[10] Ding L., Ley T.J., Larson D.E., Miller C.A., Koboldt D.C., Welch J.S., Ritchey J.K., Young M.A., Lamprecht T., McLellan M.D. (2012). Clonal evolution in relapsed acute myeloid leukaemia revealed by whole-genome sequencing. Nature.

[11] Mullighan C.G., Phillips L.A., Su X., Ma J., Miller C.B., Shurtleff S.A., Downing J.R. (2008). Genomic analysis of the clonal origins of relapsed acute lymphoblastic leukemia. Science.

[12] Keating M.J., O’Brien S., Albitar M., Lerner S., Plunkett W., Giles F., Andreeff M., Cortes J., Faderl S., Thomas D. (2005). Early results of a chemoimmunotherapy regimen of fludarabine, cyclophosphamide, and rituximab as initial therapy for chronic lymphocytic leukemia. J. Clin. Oncol..

[13] Hallek M., Fischer K., Fingerle-Rowson G., Am F., Busch R., Mayer J., Hensel M., Hopfinger G., Hess G., von Grünhagen U. (2010). Addition of rituximab to fludarabine and cyclophosphamide in patients with chronic lymphocytic leukaemia: A randomised, open-label, phase 3 trial. Lancet.

[14] Goede V., Fischer K., Busch R., Engelke A., Eichhorst B., Wendtner C.M., Chagorova T., de La Serna J., Dilhuydy M.-S., Illmer T. (2014). Obinutuzumab plus chlorambucil in patients with CLL and coexisting conditions. N. Engl. J. Med..

[15] Jessy T. (2011). Immunity over inability: The spontaneous regression of cancer. J. Nat. Sci. Biol. Med..

[16] Horowitz M.M., Gale R.P., Sondel P.M., Goldman J.M., Kersey J., Kolb H.J., Rimm A.A., Ringdén O., Rozman C., Speck B. (1990). Graft-versus-leukemia reactions after bone marrow transplantation. Blood.

[17] Dreger P., Corradini P., Kimby E., Michallet M., Milligan D., Schetelig J., Wiktor-Jedrzejczak W., Niederwieser D., Hallek M., Montserrat E. (2007). Indications for allogeneic stem cell transplantation in chronic lymphocytic leukemia: The EBMT transplant consensus. Leukemia.

[18] Kiladjian J.-J., Giraudier S., Cassinat B. (2016). Interferon-alpha for the therapy of myeloproliferative neoplasms: Targeting the malignant clone. Leukemia.

[19] Larkin J., Chiarion-Sileni V., Gonzalez R., Grob J.J., Cowey C.L., Lao C.D., Schadendorf D., Dummer R., Smylie M., Rutkowski P. (2015). Combined Nivolumab and Ipilimumab or Monotherapy in Untreated Melanoma. N. Engl. J. Med..

[20] Bauml J., Seiwert T.Y., Pfister D.G., Worden F., Liu S.V., Gilbert J., Saba N.F., Weiss J., Wirth L., Sukari A. (2017). Pembrolizumab for Platinum- and Cetuximab-Refractory Head and Neck Cancer: Results from a Single-Arm, Phase II Study. J. Clin. Oncol..

[21] Motzer R.J., Tannir N.M., McDermott D.F., Arén Frontera O., Melichar B., Choueiri T.K., Plimack E.R., Barthélémy P., Porta C., George S. (2018). Nivolumab plus Ipilimumab versus Sunitinib in Advanced Renal-Cell Carcinoma. N. Engl. J. Med..

[22] Overman M.J., Lonardi S., Wong K.Y.M., Lenz H.-J., Gelsomino F., Aglietta M., Morse M.A., van Cutsem E., McDermott R., Hill A. (2018). Durable Clinical Benefit with Nivolumab Plus Ipilimumab in DNA Mismatch Repair-Deficient/Microsatellite Instability-High Metastatic Colorectal Cancer. J. Clin. Oncol..

[23] Reck M., Rodríguez-Abreu D., Robinson A.G., Hui R., Csőszi T., Fülöp A., Gottfried M., Peled N., Tafreshi A., Cuffe S. (2016). Pembrolizumab versus Chemotherapy for PD-L1-Positive Non-Small-Cell Lung Cancer. N. Engl. J. Med..

[24] Rittmeyer A., Barlesi F., Waterkamp D., Park K., Ciardiello F., von Pawel J., Gadgeel S.M., Hida T., Kowalski D.M., Dols M.C. (2017). Atezolizumab versus docetaxel in patients with previously treated non-small-cell lung cancer (OAK): A phase 3, open-label, multicentre randomised controlled trial. Lancet.

[25] Bashey A., Medina B., Corringham S., Pasek M., Carrier E., Vrooman L., Lowy I., Solomon S.R., Morris L.E., Holland H.K. (2009). CTLA4 blockade with ipilimumab to treat relapse of malignancy after allogeneic hematopoietic cell transplantation. Blood.

[26] Daver N., Garcia-Manero G., Basu S., Boddu P.C., Alfayez M., Cortes J.E., Konopleva M., Ravandi-Kashani F., Jabbour E., Kadia T. (2018). Efficacy, Safety, and Biomarkers of Response to Azacitidine and Nivolumab in Relapsed/Refractory Acute Myeloid Leukemia: A Nonrandomized, Open-Label, Phase II Study. Cancer Discov..

[27] Carbone D.P., Reck M., Paz-Ares L., Creelan B., Horn L., Steins M., Felip E., van den Heuvel M.M., Ciuleanu T.-E., Badin F. (2017). First-Line Nivolumab in Stage IV or Recurrent Non-Small-Cell Lung Cancer. N. Engl. J. Med..

[28] Lee C.K., Man J., Lord S., Links M., Gebski V., Mok T., Yang J.C.-H. (2017). Checkpoint Inhibitors in Metastatic EGFR-Mutated Non-Small Cell Lung Cancer-A Meta-Analysis. J. Thorac. Oncol..

[29] Shitara K., Özgüroğlu M., Bang Y.-J., Di Bartolomeo M., Mandalà M., Ryu M.-H., Fornaro L., Olesiński T., Caglevic C., Chung H.C. (2018). Pembrolizumab versus paclitaxel for previously treated, advanced gastric or gastro-oesophageal junction cancer (KEYNOTE-061): A randomised, open-label, controlled, phase 3 trial. Lancet.

[30] Zeidan A.M., Knaus H.A., Robinson T.M., Towlerton A.M.H., Warren E.H., Zeidner J.F., Blackford A.L., Duffield A.S., Rizzieri D., Frattini M.G. (2018). A Multi-center Phase I Trial of Ipilimumab in Patients with Myelodysplastic Syndromes following Hypomethylating Agent Failure. Clin. Cancer Res..

[31] Herbst R.S., Baas P., Kim D.-W., Felip E., Pérez-Gracia J.L., Han J.-Y., Molina J., Kim J.-H., Arvis C.D., Ahn M.-J. (2016). Pembrolizumab versus docetaxel for previously treated, PD-L1-positive, advanced non-small-cell lung cancer (KEYNOTE-010): A randomised controlled trial. Lancet.

[32] Ciombor K.K., Goldberg R.M. (2018). Hypermutated Tumors and Immune Checkpoint Inhibition. Drugs.

[33] Zhou Y., Chen C., Zhang X., Fu S., Xue C., Ma Y., Fang W., Yang Y., Hou X., Huang Y. (2018). Immune-checkpoint inhibitor plus chemotherapy versus conventional chemotherapy for first-line treatment in advanced non-small cell lung carcinoma: A systematic review and meta-analysis. J. Immunother. Cancer.

[34] Hofmann M., Große-Hovest L., Nübling T., Pyż E., Bamberg M.L., Aulwurm S., Bühring H.-J., Schwartz K., Haen S.P., Schilbach K. (2012). Generation, selection and preclinical characterization of an Fc-optimized FLT3 antibody for the treatment of myeloid leukemia. Leukemia.

[35] He S.Z., Busfield S., Ritchie D.S., Hertzberg M.S., Durrant S., Lewis I.D., Marlton P., McLachlan A.J., Kerridge I., Bradstock K.F. (2015). A Phase 1 study of the safety, pharmacokinetics and anti-leukemic activity of the anti-CD123 monoclonal antibody CSL360 in relapsed, refractory or high-risk acute myeloid leukemia. Leuk. Lymphoma.

[36] Friedrich M., Henn A., Raum T., Bajtus M., Matthes K., Hendrich L., Wahl J., Hoffmann P., Kischel R., Kvesic M. (2014). Preclinical characterization of AMG 330, a CD3/CD33-bispecific T-cell-engaging antibody with potential for treatment of acute myelogenous leukemia. Mol. Cancer Ther..

[37] Chichili G.R., Huang L., Li H., Burke S., He L., Tang Q., Jin L., Gorlatov S., Ciccarone V., Chen F. (2015). A CD3xCD123 bispecific DART for redirecting host T cells to myelogenous leukemia: Preclinical activity and safety in nonhuman primates. Sci. Transl. Med..

[38] Larson R.A., Sievers E.L., Stadtmauer E.A., Löwenberg B., Estey E.H., Dombret H., Theobald M., Voliotis D., Bennett J.M., Richie M. (2005). Final report of the efficacy and safety of gemtuzumab ozogamicin (Mylotarg) in patients with CD33-positive acute myeloid leukemia in first recurrence. Cancer.

[39] Maude S.L., Frey N., Shaw P.A., Aplenc R., Barrett D.M., Bunin N.J., Chew A., Gonzalez V.E., Zheng Z., Lacey S.F. (2014). Chimeric antigen receptor T cells for sustained remissions in leukemia. N. Engl. J. Med..

[40] Zah E., Lin M.-Y., Silva-Benedict A., Jensen M.C., Chen Y.Y. (2016). T Cells Expressing CD19/CD20 Bispecific Chimeric Antigen Receptors Prevent Antigen Escape by Malignant B Cells. Cancer Immunol. Res..

[41] Gill S., Tasian S.K., Ruella M., Shestova O., Li Y., Porter D.L., Carroll M., Danet-Desnoyers G., Scholler J., Grupp S.A. (2014). Preclinical targeting of human acute myeloid leukemia and myeloablation using chimeric antigen receptor-modified T cells. Blood.

[42] Turazzi N., Fazio G., Rossi V., Rolink A., Cazzaniga G., Biondi A., Magnani C.F., Biagi E. (2018). Engineered T cells towards TNFRSF13C (BAFFR): A novel strategy to efficiently target B-cell acute lymphoblastic leukaemia. Br. J. Haematol..

[43] Giordano Attianese G.M.P., Marin V., Hoyos V., Savoldo B., Pizzitola I., Tettamanti S., Agostoni V., Parma M., Ponzoni M., Bertilaccio M.T.S. (2011). In vitro and in vivo model of a novel immunotherapy approach for chronic lymphocytic leukemia by anti-CD23 chimeric antigen receptor. Blood.

[44] Schiffer S., Rosinke R., Jost E., Hehmann-Titt G., Huhn M., Melmer G., Barth S., Thepen T. (2014). Targeted ex vivo reduction of CD64-positive monocytes in chronic myelomonocytic leukemia and acute myelomonocytic leukemia using human granzyme B-based cytolytic fusion proteins. Int. J. Cancer.

[45] Koerner S.P., André M.C., Leibold J.S., Kousis P.C., Kübler A., Pal M., Haen S.P., Bühring H.-J., Grosse-Hovest L., Jung G. (2017). An Fc-optimized CD133 antibody for induction of NK cell reactivity against myeloid leukemia. Leukemia.

[46] Wang J., Chen S., Xiao W., Li W., Wang L., Yang S., Wang W., Xu L., Liao S., Liu W. (2018). CAR-T cells targeting CLL-1 as an approach to treat acute myeloid leukemia. J. Hematol. Oncol..

[47] Daneshmanesh A.H., Hojjat-Farsangi M., Khan A.S., Jeddi-Tehrani M., Akhondi M.M., Bayat A.A., Ghods R., Mahmoudi A.-R., Hadavi R., Österborg A. (2012). Monoclonal antibodies against ROR1 induce apoptosis of chronic lymphocytic leukemia (CLL) cells. Leukemia.

[48] Vera J., Savoldo B., Vigouroux S., Biagi E., Pule M., Rossig C., Wu J., Heslop H.E., Rooney C.M., Brenner M.K. (2006). T lymphocytes redirected against the kappa light chain of human immunoglobulin efficiently kill mature B lymphocyte-derived malignant cells. Blood.

[49] Nadler L.M., Anderson K.C., Marti G., Bates M., Park E., Daley J.F., Schlossman S.F. (1983). B4, a human B lymphocyte-associated antigen expressed on normal, mitogen-activated, and malignant B lymphocytes. J. Immunol..

[50] Porter D.L., Hwang W.-T., Frey N.V., Lacey S.F., Shaw P.A., Loren A.W., Bagg A., Marcucci K.T., Shen A., Gonzalez V. (2015). Chimeric antigen receptor T cells persist and induce sustained remissions in relapsed refractory chronic lymphocytic leukemia. Sci. Transl. Med..

[51] Topp M.S., Gökbuget N., Stein A.S., Zugmaier G., O’Brien S., Bargou R.C., Dombret H., Fielding A.K., Heffner L., Larson R.A. (2015). Safety and activity of blinatumomab for adult patients with relapsed or refractory B-precursor acute lymphoblastic leukaemia: A multicentre, single-arm, phase 2 study. Lancet Oncol..

[52] Turtle C.J., Hanafi L.-A., Berger C., Gooley T.A., Cherian S., Hudecek M., Sommermeyer D., Melville K., Pender B., Budiarto T.M. (2016). CD19 CAR-T cells of defined CD4+:CD8+ composition in adult B cell ALL patients. J. Clin. Investig..

[53] Gust J., Hay K.A., Hanafi L.-A., Li D., Myerson D., Gonzalez-Cuyar L.F., Yeung C., Liles W.C., Wurfel M., Lopez J.A. (2017). Endothelial Activation and Blood-Brain Barrier Disruption in Neurotoxicity after Adoptive Immunotherapy with CD19 CAR-T Cells. Cancer Discov..

[54] Sotillo E., Barrett D.M., Black K.L., Bagashev A., Oldridge D., Wu G., Sussman R., Lanauze C., Ruella M., Gazzara M.R. (2015). Convergence of Acquired Mutations and Alternative Splicing of CD19 Enables Resistance to CART-19 Immunotherapy. Cancer Discov..

[55] Braig F., Brandt A., Goebeler M., Tony H.-P., Kurze A.-K., Nollau P., Bumm T., Böttcher S., Bargou R.C., Binder M. (2017). Resistance to anti-CD19/CD3 BiTE in acute lymphoblastic leukemia may be mediated by disrupted CD19 membrane trafficking. Blood.

[56] Qin H., Ramakrishna S., Nguyen S., Fountaine T.J., Ponduri A., Stetler-Stevenson M., Yuan C.M., Haso W., Shern J.F., Shah N.N. (2018). Preclinical Development of Bivalent Chimeric Antigen Receptors Targeting Both CD19 and CD22. Mol. Ther. Oncolytics.

[57] Shan D., Press O.W. (1995). Constitutive endocytosis and degradation of CD22 by human B cells. J Immunol..

[58] Kantarjian H.M., DeAngelo D.J., Stelljes M., Martinelli G., Liedtke M., Stock W., Gökbuget N., O’Brien S., Wang K., Wang T. (2016). Inotuzumab Ozogamicin versus Standard Therapy for Acute Lymphoblastic Leukemia. N. Engl. J. Med..

[59] Fry T.J., Shah N.N., Orentas R.J., Stetler-Stevenson M., Yuan C.M., Ramakrishna S., Wolters P., Martin S., Delbrook C., Yates B. (2017). CD22-targeted CAR T cells induce remission in B-ALL that is naive or resistant to CD19-targeted CAR immunotherapy. Nat. Med..

[60] Jilani I., Estey E., Huh Y., Joe Y., Manshouri T., Yared M., Giles F., Kantarjian H., Cortes J., Thomas D. (2002). Differences in CD33 intensity between various myeloid neoplasms. Am. J. Clin. Pathol..

[61] Taussig D.C., Pearce D.J., Simpson C., Rohatiner A.Z., Lister T.A., Kelly G., Luongo J.L., Danet-Desnoyers G.-A.H., Bonnet D. (2005). Hematopoietic stem cells express multiple myeloid markers: Implications for the origin and targeted therapy of acute myeloid leukemia. Blood.

[62] Wang Q.-S., Wang Y., Lv H.-Y., Han Q.-W., Fan H., Guo B., Wang L.-L., Han W.-D. (2015). Treatment of CD33-directed chimeric antigen receptor-modified T cells in one patient with relapsed and refractory acute myeloid leukemia. Mol. Ther..

[63] Petersdorf S.H., Kopecky K.J., Slovak M., Willman C., Nevill T., Brandwein J., Larson R.A., Erba H.P., Stiff P.J., Stuart R.K. (2013). A phase 3 study of gemtuzumab ozogamicin during induction and postconsolidation therapy in younger patients with acute myeloid leukemia. Blood.

[64] Baron J., Wang E.S. (2018). Gemtuzumab ozogamicin for the treatment of acute myeloid leukemia. Expert Rev. Clin. Pharmacol..

[65] Kenderian S.S., Ruella M., Shestova O., Klichinsky M., Aikawa V., Morrissette J.J.D., Scholler J., Song D., Porter D.L., Carroll M. (2015). CD33-specific chimeric antigen receptor T cells exhibit potent preclinical activity against human acute myeloid leukemia. Leukemia.

[66] Muñoz L., Nomdedéu J.F., López O., Carnicer M.J., Bellido M., Aventín A., Brunet S., Sierra J. (2001). Interleukin-3 receptor alpha chain (CD123) is widely expressed in hematologic malignancies. Haematologica.

[67] Jordan C.T., Upchurch D., Szilvassy S.J., Guzman M.L., Howard D.S., Pettigrew A.L., Meyerrose T., Rossi R., Grimes B., Rizzieri D.A. (2000). The interleukin-3 receptor alpha chain is a unique marker for human acute myelogenous leukemia stem cells. Leukemia.

[68] Pizzitola I., Anjos-Afonso F., Rouault-Pierre K., Lassailly F., Tettamanti S., Spinelli O., Biondi A., Biagi E., Bonnet D. (2014). Chimeric antigen receptors against CD33/CD123 antigens efficiently target primary acute myeloid leukemia cells in vivo. Leukemia.

[69] Busfield S.J., Biondo M., Wong M., Ramshaw H.S., Lee E.M., Ghosh S., Braley H., Panousis C., Roberts A.W., He S.Z. (2014). Targeting of acute myeloid leukemia in vitro and in vivo with an anti-CD123 mAb engineered for optimal ADCC. Leukemia.

[70] Ruella M., Barrett D.M., Kenderian S.S., Shestova O., Hofmann T.J., Perazzelli J., Klichinsky M., Aikawa V., Nazimuddin F., Kozlowski M. (2016). Dual CD19 and CD123 targeting prevents antigen-loss relapses after CD19-directed immunotherapies. J. Clin. Investig..

[71] Adolfsson J., Borge O.J., Bryder D., Theilgaard-Mönch K., Astrand-Grundström I., Sitnicka E., Sasaki Y., Jacobsen S.E. (2001). Upregulation of Flt3 expression within the bone marrow Lin(-)Sca1(+)c-kit(+) stem cell compartment is accompanied by loss of self-renewal capacity. Immunity.

[72] Santos F.P.S., Jones D., Qiao W., Cortes J.E., Ravandi F., Estey E.E., Verma D., Kantarjian H., Borthakur G. (2011). Prognostic value of FLT3 mutations among different cytogenetic subgroups in acute myeloid leukemia. Cancer.

[73] Chan P.M. (2011). Differential signaling of Flt3 activating mutations in acute myeloid leukemia: A working model. Protein Cell.

[74] Jetani H., Garcia-Cadenas I., Nerreter T., Thomas S., Rydzek J., Meijide J.B., Bonig H., Herr W., Sierra J., Einsele H. (2018). CAR T-cells targeting FLT3 have potent activity against FLT3-ITD+ AML and act synergistically with the FLT3-inhibitor crenolanib. Leukemia.

[75] Durben M., Schmiedel D., Hofmann M., Vogt F., Nübling T., Pyz E., Bühring H.-J., Rammensee H.-G., Salih H.R., Große-Hovest L. (2015). Characterization of a bispecific FLT3 X CD3 antibody in an improved, recombinant format for the treatment of leukemia. Mol. Ther..

[76] Reiter K., Polzer H., Krupka C., Maiser A., Vick B., Rothenberg-Thurley M., Metzeler K.H., Dörfel D., Salih H.R., Jung G. (2018). Tyrosine kinase inhibition increases the cell surface localization of FLT3-ITD and enhances FLT3-directed immunotherapy of acute myeloid leukemia. Leukemia.

[77] Anguille S., van de Velde A.L., Smits E.L., van Tendeloo V.F., Juliusson G., Cools N., Nijs G., Stein B., Lion E., van Driessche A. (2017). Dendritic cell vaccination as postremission treatment to prevent or delay relapse in acute myeloid leukemia. Blood.

[78] Kranz L.M., Diken M., Haas H., Kreiter S., Loquai C., Reuter K.C., Meng M., Fritz D., Vascotto F., Hefesha H. (2016). Systemic RNA delivery to dendritic cells exploits antiviral defence for cancer immunotherapy. Nature.

[79] Maslak P.G., Dao T., Bernal Y., Chanel S.M., Zhang R., Frattini M., Rosenblat T., Jurcic J.G., Brentjens R.J., Arcila M.E. (2018). Phase 2 trial of a multivalent WT1 peptide vaccine (galinpepimut-S) in acute myeloid leukemia. Blood Adv..

[80] Dossett M.L., Teague R.M., Schmitt T.M., Tan X., Cooper L.J., Pinzon C., Greenberg P.D. (2009). Adoptive immunotherapy of disseminated leukemia with TCR-transduced, CD8+ T cells expressing a known endogenous TCR. Mol. Ther..

[81] Sergeeva A., He H., Ruisaard K., St John L., Alatrash G., Clise-Dwyer K., Li D., Patenia R., Hong R., Sukhumalchandra P. (2016). Activity of 8F4, a T-cell receptor-like anti-PR1/HLA-A2 antibody, against primary human AML in vivo. Leukemia.

[82] Sahin U., Derhovanessian E., Miller M., Kloke B.-P., Simon P., Löwer M., Bukur V., Tadmor A.D., Luxemburger U., Schrörs B. (2017). Personalized RNA mutanome vaccines mobilize poly-specific therapeutic immunity against cancer. Nature.

[83] Greiner J., Li L., Ringhoffer M., Barth T.F.E., Giannopoulos K., Guillaume P., Ritter G., Wiesneth M., Döhner H., Schmitt M. (2005). Identification and characterization of epitopes of the receptor for hyaluronic acid-mediated motility (RHAMM/CD168) recognized by CD8+ T cells of HLA-A2-positive patients with acute myeloid leukemia. Blood.

[84] Nelde A., Walz J.S., Kowalewski D.J., Schuster H., Wolz O.-O., Peper J.K., Cardona Gloria Y., Langerak A.W., Muggen A.F., Claus R. (2017). HLA class I-restricted MYD88 L265P-derived peptides as specific targets for lymphoma immunotherapy. Oncoimmunology.

[85] Weinzierl A.O., Lemmel C., Schoor O., Müller M., Krüger T., Wernet D., Hennenlotter J., Stenzl A., Klingel K., Rammensee H.-G. (2007). Distorted relation between mRNA copy number and corresponding major histocompatibility complex ligand density on the cell surface. Mol. Cell Proteomics.

[86] Berlin C., Kowalewski D.J., Schuster H., Mirza N., Walz S., Handel M., Schmid-Horch B., Salih H.R., Kanz L., Rammensee H.-G. (2015). Mapping the HLA ligandome landscape of acute myeloid leukemia: A targeted approach toward peptide-based immunotherapy. Leukemia.

[87] Bassani-Sternberg M., Bräunlein E., Klar R., Engleitner T., Sinitcyn P., Audehm S., Straub M., Weber J., Slotta-Huspenina J., Specht K. (2016). Direct identification of clinically relevant neoepitopes presented on native human melanoma tissue by mass spectrometry. Nat. Commun..

[88] Fortier M.-H., Caron E., Hardy M.-P., Voisin G., Lemieux S., Perreault C., Thibault P. (2008). The MHC class I peptide repertoire is molded by the transcriptome. J. Exp. Med..

[89] Bassani-Sternberg M., Pletscher-Frankild S., Jensen L.J., Mann M. (2015). Mass spectrometry of human leukocyte antigen class I peptidomes reveals strong effects of protein abundance and turnover on antigen presentation. Mol. Cell Proteomics.

[90] Pritchard A.L., Burel J.G., Neller M.A., Hayward N.K., Lopez J.A., Fatho M., Lennerz V., Wölfel T., Schmidt C.W. (2015). Exome Sequencing to Predict Neoantigens in Melanoma. Cancer Immunol. Res..

[91] Schumacher T.N., Schreiber R.D. (2015). Neoantigens in cancer immunotherapy. Science.

[92] Tran E., Ahmadzadeh M., Lu Y.-C., Gros A., Turcotte S., Robbins P.F., Gartner J.J., Zheng Z., Li Y.F., Ray S. (2015). Immunogenicity of somatic mutations in human gastrointestinal cancers. Science.

[93] Ott P.A., Hu Z., Keskin D.B., Shukla S.A., Sun J., Bozym D.J., Zhang W., Luoma A., Giobbie-Hurder A., Peter L. (2017). An immunogenic personal neoantigen vaccine for patients with melanoma. Nature.

[94] Snyder A., Makarov V., Merghoub T., Yuan J., Zaretsky J.M., Desrichard A., Walsh L.A., Postow M.A., Wong P., Ho T.S. (2014). Genetic basis for clinical response to CTLA-4 blockade in melanoma. N. Engl. J. Med..

[95] Gubin M.M., Zhang X., Schuster H., Caron E., Ward J.P., Noguchi T., Ivanova Y., Hundal J., Arthur C.D., Krebber W.-J. (2014). Checkpoint blockade cancer immunotherapy targets tumour-specific mutant antigens. Nature.

[96] Finn O.J., Rammensee H.-G. (2018). Is It Possible to Develop Cancer Vaccines to Neoantigens, What Are the Major Challenges, and How Can These Be Overcome? Neoantigens: Nothing New in Spite of the Name. Cold Spring Harb. Perspect. Biol..

[97] Yadav M., Jhunjhunwala S., Phung Q.T., Lupardus P., Tanguay J., Bumbaca S., Franci C., Cheung T.K., Fritsche J., Weinschenk T. (2014). Predicting immunogenic tumour mutations by combining mass spectrometry and exome sequencing. Nature.

[98] Hilf N., Kuttruff-Coqui S., Frenzel K., Bukur V., Stevanović S., Gouttefangeas C., Platten M., Tabatabai G., Dutoit V., van der Burg S.H. (2019). Actively personalized vaccination trial for newly diagnosed glioblastoma. Nature.

[99] Di Marco M., Peper J.K., Rammensee H.-G. (2017). Identification of Immunogenic Epitopes by MS/MS. Cancer J..

[100] Nelde A., Kowalewski D.J., Backert L., Schuster H., Werner J.-O., Klein R., Kohlbacher O., Kanz L., Salih H.R., Rammensee H.-G. (2018). HLA ligandome analysis of primary chronic lymphocytic leukemia (CLL) cells under lenalidomide treatment confirms the suitability of lenalidomide for combination with T-cell-based immunotherapy. Oncoimmunology.

[101] Schuster H., Peper J.K., Bösmüller H.-C., Röhle K., Backert L., Bilich T., Ney B., Löffler M.W., Kowalewski D.J., Trautwein N. (2017). The immunopeptidomic landscape of ovarian carcinomas. Proc. Natl. Acad. Sci. USA.

[102] Stickel J.S., Weinzierl A.O., Hillen N., Drews O., Schuler M.M., Hennenlotter J., Wernet D., Müller C.A., Stenzl A., Rammensee H.-G. (2009). HLA ligand profiles of primary renal cell carcinoma maintained in metastases. Cancer Immunol. Immunother..

[103] Kowalewski D.J., Schuster H., Backert L., Berlin C., Kahn S., Kanz L., Salih H.R., Rammensee H.-G., Stevanovic S., Stickel J.S. (2015). HLA ligandome analysis identifies the underlying specificities of spontaneous antileukemia immune responses in chronic lymphocytic leukemia (CLL). Proc. Natl. Acad. Sci. USA.

[104] Bilich T., Nelde A., Bichmann L., Roerden M., Salih H.R., Kowalewski D.J., Schuster H., Tsou C.-C., Marcu A., Neidert M.C. (2019). The HLA ligandome landscape of chronic myeloid leukemia delineates novel T-cell epitopes for immunotherapy. Blood.

[105] Walz S., Stickel J.S., Kowalewski D.J., Schuster H., Weisel K., Backert L., Kahn S., Nelde A., Stroh T., Handel M. (2015). The antigenic landscape of multiple myeloma: Mass spectrometry (re)defines targets for T-cell-based immunotherapy. Blood.

[106] Heidenreich F., Rücker-Braun E., Walz J.S., Eugster A., Kühn D., Dietz S., Nelde A., Tunger A., Wehner R., Link C.S. (2017). Mass spectrometry-based identification of a naturally presented receptor tyrosine kinase-like orphan receptor 1-derived epitope recognized by CD8+ cytotoxic T cells. Haematologica.

[107] Walz J.S., Kowalewski D.J., Backert L., Nelde A., Kohlbacher O., Weide B., Kanz L., Salih H.R., Rammensee H.-G., Stevanović S. (2018). Favorable immune signature in CLL patients, defined by antigen-specific T-cell responses, might prevent second skin cancers. Leuk. Lymphoma.

[108] Kowalewski D.J., Stevanovic S., Rammensee H.-G., Stickel J.S. (2015). Antileukemia T-cell responses in CLL—We don’t need no aberration. Oncoimmunology.

[109] Chong C., Marino F., Pak H., Racle J., Daniel R.T., Müller M., Gfeller D., Coukos G., Bassani-Sternberg M. (2018). High-throughput and Sensitive Immunopeptidomics Platform Reveals Profound Interferonγ-Mediated Remodeling of the Human Leukocyte Antigen (HLA) Ligandome. Mol. Cell Proteomics.

[110] Singh-Jasuja H., Emmerich N.P.N., Rammensee H.-G. (2004). The Tübingen approach: Identification, selection, and validation of tumor-associated HLA peptides for cancer therapy. Cancer Immunol. Immunother..

[111] Löffler M.W., Chandran P.A., Laske K., Schroeder C., Bonzheim I., Walzer M., Hilke F.J., Trautwein N., Kowalewski D.J., Schuster H. (2016). Personalized peptide vaccine-induced immune response associated with long-term survival of a metastatic cholangiocarcinoma patient. J. Hepatol..

[112] Backert L., Kowalewski D.J., Walz S., Schuster H., Berlin C., Neidert M.C., Schemionek M., Brümmendorf T.H., Vucinic V., Niederwieser D. (2017). A meta-analysis of HLA peptidome composition in different hematological entities: Entity-specific dividing lines and “pan-leukemia” antigens. Oncotarget.

[113] Miwa H., Beran M., Saunders G.F. (1992). Expression of the Wilms’ tumor gene (WT1) in human leukemias. Leukemia.

[114] Inoue K., Ogawa H., Sonoda Y., Kimura T., Sakabe H., Oka Y., Miyake S., Tamaki H., Oji Y., Yamagami T. (1997). Aberrant overexpression of the Wilms tumor gene (WT1) in human leukemia. Blood.

[115] Yoon J.-H., Kim H.-J., Kwak D.-H., Park S.-S., Jeon Y.-W., Lee S.-E., Cho B.-S., Eom K.-S., Kim Y.-J., Lee S. (2017). High WT1 expression is an early predictor for relapse in patients with acute promyelocytic leukemia in first remission with negative PML-RARa after anthracycline-based chemotherapy: A single-center cohort study. J. Hematol. Oncol..

[116] Candoni A., de Marchi F., Zanini F., Zannier M.E., Simeone E., Toffoletti E., Chiarvesio A., Cerno M., Filì C., Patriarca F. (2017). Predictive value of pretransplantation molecular minimal residual disease assessment by WT1 gene expression in FLT3-positive acute myeloid leukemia. Exp. Hematol..

[117] Cheever M.A., Allison J.P., Ferris A.S., Finn O.J., Hastings B.M., Hecht T.T., Mellman I., Prindiville S.A., Viner J.L., Weiner L.M. (2009). The prioritization of cancer antigens: A national cancer institute pilot project for the acceleration of translational research. Clin. Cancer Res..

[118] Di Stasi A., Jimenez A.M., Minagawa K., Al-Obaidi M., Rezvani K. (2015). Review of the Results of WT1 Peptide Vaccination Strategies for Myelodysplastic Syndromes and Acute Myeloid Leukemia from Nine Different Studies. Front. Immunol..

[119] Oka Y., Tsuboi A., Nakata J., Nishida S., Hosen N., Kumanogoh A., Oji Y., Sugiyama H. (2017). Wilms’ Tumor Gene 1 (WT1) Peptide Vaccine Therapy for Hematological Malignancies: From CTL Epitope Identification to Recent Progress in Clinical Studies Including a Cure-Oriented Strategy. Oncol. Res. Treat..

[120] Nakata J., Nakae Y., Kawakami M., Morimoto S., Motooka D., Hosen N., Fujiki F., Nakajima H., Hasegawa K., Nishida S. (2018). Wilms tumour 1 peptide vaccine as a cure-oriented post-chemotherapy strategy for patients with acute myeloid leukaemia at high risk of relapse. Br. J. Haematol..

[121] Kim H.-J., Sohn H.-J., Hong J.-A., Lee H.-J., Sohn D.-H., Shin C.-A., Cho H.-I., Min W.-S., Kim T.-G. (2018). Post-transplant immunotherapy with WT1-specific CTLs for high-risk acute myelogenous leukemia: A prospective clinical phase I/II trial. Bone Marrow Transplant..

[122] Dagvadorj N., Deuretzbacher A., Weisenberger D., Baumeister E., Trebing J., Lang I., Köchel C., Kapp M., Kapp K., Beilhack A. (2017). Targeting of the WT191-138 fragment to human dendritic cells improves leukemia-specific T-cell responses providing an alternative approach to WT1-based vaccination. Cancer Immunol. Immunother..

[123] Jafri M.A., Ansari S.A., Alqahtani M.H., Shay J.W. (2016). Roles of telomeres and telomerase in cancer, and advances in telomerase-targeted therapies. Genome Med..

[124] Vonderheide R.H., Hahn W.C., Schultze J.L., Nadler L.M. (1999). The Telomerase Catalytic Subunit Is a Widely Expressed Tumor-Associated Antigen Recognized by Cytotoxic T Lymphocytes. Immunity.

[125] Khoury H.J., Collins R.H., Blum W., Stiff P.S., Elias L., Lebkowski J.S., Reddy A., Nishimoto K.P., Sen D., Wirth E.D. (2017). Immune responses and long-term disease recurrence status after telomerase-based dendritic cell immunotherapy in patients with acute myeloid leukemia. Cancer.

[126] Sandri S., Bobisse S., Moxley K., Lamolinara A., de Sanctis F., Boschi F., Sbarbati A., Fracasso G., Ferrarini G., Hendriks R.W. (2016). Feasibility of Telomerase-Specific Adoptive T-cell Therapy for B-cell Chronic Lymphocytic Leukemia and Solid Malignancies. Cancer Res..

[127] Sandri S., de Sanctis F., Lamolinara A., Boschi F., Poffe O., Trovato R., Fiore A., Sartori S., Sbarbati A., Bondanza A. (2017). Effective control of acute myeloid leukaemia and acute lymphoblastic leukaemia progression by telomerase specific adoptive T-cell therapy. Oncotarget.

[128] Greiner J., Ringhoffer M., Taniguchi M., Schmitt A., Kirchner D., Krähn G., Heilmann V., Gschwend J., Bergmann L., Döhner H. (2002). Receptor for hyaluronan acid–mediated motility (RHAMM) is a new immunogenic leukemia-associated antigen in acute and chronic myeloid leukemia. Exp. Hematol..

[129] Giannopoulos K., Li L., Bojarska-Junak A., Rolinski J., Dmoszynska A., Hus I., Greiner J., Renner C., Döhner H., Schmitt M. (2006). Expression of RHAMM/CD168 and other tumor-associated antigens in patients with B-cell chronic lymphocytic leukemia. Int. J. Oncol..

[130] Shalini C.N.S., Suman F.R., Jacob J.S., Rajendran R., Scott J.X., Latha M.S. (2018). Prognostic significance of receptor for hyaluronan acid-mediated motility (CD168) in acute pediatric leukemias—Assessment of clinical outcome, post induction, end of treatment and minimal residual disease. Hematol. Transfus. Cell Ther..

[131] Schmitt M., Schmitt A., Rojewski M.T., Chen J., Giannopoulos K., Fei F., Yu Y., Götz M., Heyduk M., Ritter G. (2008). RHAMM-R3 peptide vaccination in patients with acute myeloid leukemia, myelodysplastic syndrome, and multiple myeloma elicits immunologic and clinical responses. Blood.

[132] Giannopoulos K., Dmoszynska A., Kowal M., Rolinski J., Gostick E., Price D.A., Greiner J., Rojewski M., Stilgenbauer S., Döhner H. (2010). Peptide vaccination elicits leukemia-associated antigen-specific cytotoxic CD8+ T-cell responses in patients with chronic lymphocytic leukemia. Leukemia.

[133] Willemen Y., van den Bergh J.M.J., Bonte S.M., Anguille S., Heirman C., Stein B.M.H., Goossens H., Kerre T., Thielemans K., Peeters M. (2016). The tumor-associated antigen RHAMM (HMMR/CD168) is expressed by monocyte-derived dendritic cells and presented to T cells. Oncotarget.

[134] Snauwaert S., Vanhee S., Goetgeluk G., Verstichel G., van Caeneghem Y., Velghe I., Philippé J., Berneman Z.N., Plum J., Taghon T. (2012). RHAMM/HMMR (CD168) is not an ideal target antigen for immunotherapy of acute myeloid leukemia. Haematologica.

[135] Rezvani K., Yong A.S.M., Tawab A., Jafarpour B., Eniafe R., Mielke S., Savani B.N., Keyvanfar K., Li Y., Kurlander R. (2009). Ex vivo characterization of polyclonal memory CD8+ T-cell responses to PRAME-specific peptides in patients with acute lymphoblastic leukemia and acute and chronic myeloid leukemia. Blood.

[136] Chang A.Y., Dao T., Gejman R.S., Jarvis C.A., Scott A., Dubrovsky L., Mathias M.D., Korontsvit T., Zakhaleva V., Curcio M. (2017). A therapeutic T cell receptor mimic antibody targets tumor-associated PRAME peptide/HLA-I antigens. J. Clin. Investig..

[137] Matko S., Manderla J., Bonsack M., Schmitz M., Bornhauser M., Tonn T., Odendahl M. (2018). PRAME peptide-specific CD8+ T cells represent the predominant response against leukemia-associated antigens in healthy individuals. Eur. J. Immunol..

[138] Molldrem J., Dermime S., Parker K., Jiang Y.Z., Mavroudis D., Hensel N., Fukushima P., Barrett A.J. (1996). Targeted T-cell therapy for human leukemia: Cytotoxic T lymphocytes specific for a peptide derived from proteinase 3 preferentially lyse human myeloid leukemia cells. Blood.

[139] Qazilbash M.H., Wieder E., Thall P.F., Wang X., Rios R., Lu S., Kanodia S., Ruisaard K.E., Giralt S.A., Estey E.H. (2017). PR1 peptide vaccine induces specific immunity with clinical responses in myeloid malignancies. Leukemia.

[140] Sergeeva A., Alatrash G., He H., Ruisaard K., Lu S., Wygant J., McIntyre B.W., Ma Q., Li D., St John L. (2011). An anti-PR1/HLA-A2 T-cell receptor-like antibody mediates complement-dependent cytotoxicity against acute myeloid leukemia progenitor cells. Blood.

[141] Herrmann A.C., Im J.S., Pareek S., Ruiz-Vasquez W., Lu S., Sergeeva A., Mehrens J., He H., Alatrash G., Sukhumalchandra P. (2018). A Novel T-Cell Engaging Bi-specific Antibody Targeting the Leukemia Antigen PR1/HLA-A2. Front. Immunol..

[142] Ma Q., Garber H.R., Lu S., He H., Tallis E., Ding X., Sergeeva A., Wood M.S., Dotti G., Salvado B. (2016). A novel TCR-like CAR with specificity for PR1/HLA-A2 effectively targets myeloid leukemia in vitro when expressed in human adult peripheral blood and cord blood T cells. Cytotherapy.

[143] Mori A., Wada H., Nishimura Y., Okamoto T., Takemoto Y., Kakishita E. (2002). Expression of the Antiapoptosis Gene Survivin in Human Leukemia. Int. J. Hematol..

[144] Park E., Gang E.J., Hsieh Y.-T., Schaefer P., Chae S., Klemm L., Huantes S., Loh M., Conway E.M., Kang E.-S. (2011). Targeting survivin overcomes drug resistance in acute lymphoblastic leukemia. Blood.

[145] Boullosa L.F., Savaliya P., Bonney S., Orchard L., Wickenden H., Lee C., Smits E., Banham A.H., Mills K.I., Orchard K. (2018). Identification of survivin as a promising target for the immunotherapy of adult B-cell acute lymphoblastic leukemia. Oncotarget.

[146] Comoli P., Basso S., Riva G., Barozzi P., Guido I., Gurrado A., Quartuccio G., Rubert L., Lagreca I., Vallerini D. (2017). BCR-ABL-specific T-cell therapy in Ph+ ALL patients on tyrosine-kinase inhibitors. Blood.

[147] Kessler J.H., Bres-Vloemans S.A., van Veelen P.A., de Ru A., Huijbers I.J.G., Camps M., Mulder A., Offringa R., Drijfhout J.W., Leeksma O.C. (2006). BCR-ABL fusion regions as a source of multiple leukemia-specific CD8+ T-cell epitopes. Leukemia.

[148] Ueda N., Zhang R., Tatsumi M., Liu T.-Y., Kitayama S., Yasui Y., Sugai S., Iwama T., Senju S., Okada S. (2018). BCR-ABL-specific CD4+ T-helper cells promote the priming of antigen-specific cytotoxic T cells via dendritic cells. Cell. Mol. Immunol..

[149] Cai A., Keskin D.B., DeLuca D.S., Alonso A., Zhang W., Zhang G.L., Hammond N.N., Nardi V., Stone R.M., Neuberg D. (2012). Mutated BCR-ABL generates immunogenic T-cell epitopes in CML patients. Clin. Cancer Res..

[150] Clark R.E. (2001). Direct evidence that leukemic cells present HLA-associated immunogenic peptides derived from the BCR-ABL b3a2 fusion protein. Blood.

[151] Dvorakova D., Racil Z., Jeziskova I., Palasek I., Protivankova M., Lengerova M., Razga F., Mayer J. (2010). Monitoring of minimal residual disease in acute myeloid leukemia with frequent and rare patient-specific NPM1 mutations. Am. J. Hematol..

[152] Greiner J., Ono Y., Hofmann S., Schmitt A., Mehring E., Götz M., Guillaume P., Döhner K., Mytilineos J., Döhner H. (2012). Mutated regions of nucleophosmin 1 elicit both CD4(+) and CD8(+) T-cell responses in patients with acute myeloid leukemia. Blood.

[153] Forghieri F., Riva G., Lagreca I., Barozzi P., Vallerini D., Morselli M., Paolini A., Bresciani P., Colaci E., Maccaferri M. (2019). Characterization and dynamics of specific T cells against nucleophosmin-1 (NPM1)-mutated peptides in patients with NPM1-mutated acute myeloid leukemia. Oncotarget.

[154] Kuželová K., Brodská B., Schetelig J., Röllig C., Ráčil Z., Walz J.S., Helbig G., Fuchs O., Vraná M., Pecherková P. (2018). Association of HLA class I type with prevalence and outcome of patients with acute myeloid leukemia and mutated nucleophosmin. PLoS ONE.

[155] Van der Lee D.I., Reijmers R.M., Honders M.W., Hagedoorn R.S., de Jong R.C., Kester M.G., van der Steen D.M., de Ru A.H., Kweekel C., Bijen H.M. (2019). Mutated nucleophosmin 1 as immunotherapy target in acute myeloid leukemia. J. Clin. Investig..

[156] Capitini C.M., Fry T.J., Mackall C.L. (2009). Cytokines as Adjuvants for Vaccine and Cellular Therapies for Cancer. Am. J. Immunol..

[157] Melero I., Berman D.M., Aznar M.A., Korman A.J., Pérez Gracia J.L., Haanen J. (2015). Evolving synergistic combinations of targeted immunotherapies to combat cancer. Nat. Rev. Cancer.

[158] Deres K., Schild H., Wiesmüller K.H., Jung G., Rammensee H.G. (1989). In vivo priming of virus-specific cytotoxic T lymphocytes with synthetic lipopeptide vaccine. Nature.

[159] Rammensee H.G., Chandran A., Zelba H., Gouttefangeas C., Kowalewski D., Di Marco M., Haen S., Löffler M., Klein R., Laske K. A new synthetic lipopeptide is a superior adjuvant for peptide vaccination. Presented at the 14th Annual Meeting of the Association for Cancer Immunotherapy CIMT.

[160] Temizoz B., Kuroda E., Ishii K.J. (2016). Vaccine adjuvants as potential cancer immunotherapeutics. Int. Immunol..

